# Impact of family functioning and peer pressure on nicotine dependence: a cross-sectional study among medical students in South Punjab, Pakistan

**DOI:** 10.1097/MS9.0000000000004859

**Published:** 2026-04-06

**Authors:** Fakiha Sultana Malik, Areej Imtiaz, Tayyaba Abdullah, Muddassir Khalid, Zainab Salman, Muhammad Salman Nadeem, Mahnoor Fatima, Muhammad Farhan Jamil

**Affiliations:** a Department of Medicine, Nishtar Medical University, Multan, Pakistan; b Shaheed Ziaur Rahman Medical College, Rajshahi Medical University, Bogura, Bangladesh

**Keywords:** cross-sectional, family relations, medical students, nicotine, Pakistan, peer influence, smoking, tobacco use disorder

## Abstract

**Background:**

Nicotine addiction among medical students is concerning, as these future healthcare providers are expected to model healthy lifestyle choices. While family and peer influences on smoking are well established globally, they remain underexplored in Punjab’s cultural setting. This study aims to investigate the impact of family functioning and peer pressure on nicotine dependence among medical students in South Punjab.

**Materials and methods:**

A descriptive-analytic cross-sectional study was conducted from May to July 2025 among medical students enrolled in medical colleges in South Punjab using a structured, self-administered questionnaire. Nicotine dependence was assessed using the Fagerström Test for Nicotine Dependence, family functioning using the Family Assessment Device-General Functioning subscale, and peer pressure using a structured Indicators of Peer Pressure questionnaire. This study employed convenience sampling, and the data were analyzed using descriptive statistics, chi-square tests, independent sample *t*-test, and two-way ANOVA using SPSS version 26.

**Results:**

Among 366 students, 16.1% were smokers. Family functioning and peer pressure showed a significant interaction (*P* = 0.044), and nicotine dependence was significantly higher among students from dysfunctional families who also experienced peer pressure (*P* = 0.022). Males were more likely to smoke (*P* < 0.001) and to experience peer pressure (*P* < 0.001), and peer pressure varied by year of study (*P* = 0.028).

**Conclusion:**

There is a need for targeted smoking cessation and gender-focused interventions among medical students, considering the interplay of family dysfunction and peer pressure. Further research is needed to confirm these findings and inform effective interventions.

## Introduction

The issue of nicotine dependence, defined as a chronic condition characterized by compulsive use of nicotine-containing products despite adverse health effects, is escalating, especially among young adults, including medical students, and poses a significant threat to public health, as these individuals are expected to set a positive example and promote healthy behaviors^[^1,2^]^. Despite being well informed of smoking’s adverse health effects, a notable segment of medical students continues to engage in cigarette smoking and other nicotine products such as water-pipe smoking, e-cigarettes, and chewing tobacco[3]. Of all the nicotine products, cigarette smoking is widely used and is associated with substantial health risks, and a high potential for addiction that sustains nicotine dependence. The influence of family functioning, defined as the capacity of a family to provide emotional support, maintain effective communication, and regulate the behaviors of its members, on nicotine addiction is critical, with parental smoking, family disputes, and the degree of supervision playing major roles. Low parental supervision also raises the risk of early tobacco use[4].

**HIGHLIGHTS**

- A total of 16.1% of surveyed medical students were smokers, with varying levels of nicotine dependence.
- Peer pressure significantly amplified the effect of family dysfunction on nicotine use.
- Male students were more likely to smoke and be affected by peer pressure.
- Findings emphasize the need for institutional smoking cessation programs and gender-specific approaches.

The role of family and social environment in shaping adolescent e-cigarette use is crucial, with studies demonstrating a proportionate rise in nicotine use and domestic conflicts[5]. According to a study, peer pressure, defined as the social influence exerted by an individual’s peers to encourage conformity to certain behaviors or values, including initiation or continuation of nicotine use, hostel life, and social acceptance, emerged as major contributors to nicotine initiation among medical students^[^6,7^]^. Among medical students, peer influence significantly encourages nicotine use and addiction, as many smoke to gain social acceptance and approval[8]. The competitive and stressful medical school environment often leads students to use smoking as a stress relief strategy, reinforced by their peers’ habits. This dynamic normalizes smoking and creates barriers to quitting, highlighting the importance of addressing peer influence in prevention strategies[9]. A comprehensive analysis involving 107 527 student health professionals across 70 countries with response rates ranging from 40% to 100% found that nicotine intake among medical students was highest in European countries (20%) and the Americas (13%)[10]. Similarly, another study identified peer pressure as the most common determinant (57.69%) for developing smoking habits among medical students[11]. In Pakistan, 10.7% of final year medical students smoke[12].

Nicotine addiction among medical students poses a serious public health concern, affecting both their well-being and professional responsibilities[13]. Few studies explore the combined impact of family and peer influences on health behaviors, although this interaction is crucial for preventing addiction[14]. Nicotine addiction is well studied in the general population but overlooked among medical students. Exploring the role of family and peer relationships in nicotine dependence is essential for identifying the social drivers of tobacco use[15]. Moreover, the economic burden of treating nicotine-related health conditions, including long-term management of cardiovascular, respiratory, and oncological diseases, places significant strain on healthcare systems[16]. Addressing nicotine dependence early can reduce future healthcare costs and prevent long-term consequences not only for the individual but equally for healthcare services and the broader community. This understanding can guide creation of focused prevention strategies and informed policy measures to address these influences, ultimately aiding in reducing the nicotine addiction and promote better health outcomes.

## Materials and methods

This cross-sectional study was carried out in the medical colleges of South Punjab from May to July 2025. Ethical approval was obtained from the Institutional Ethical Review Board of a medical university in Multan, Pakistan. All procedures in this study were conducted in accordance with the International Ethical Guidelines for Human Research in Health Care and the Helsinki Declaration (2013 Revision) (2016). The study ensured that subjects participated on a voluntary and anonymous basis, and informed consent was secured from each participant.

Students enrolled in these colleges who consented to participate were included in the study, and those who refused consent, were absent, or were uncooperative were excluded. Sample size was calculated using OpenEpi v3.01, assuming a 95% confidence level (*z* = 1.96), 5% margin of error, and a conservative prevalence estimate of 50% (*P* = 0.5) to maximize sample size. This resulted in a required sample size of 366, which provides approximately 100% power to estimate prevalence within a 5% margin of error, ensuring adequate precision and reliability. Convenience sampling was used, in which participants were selected based on their accessibility and availability. Although this approach was non-random, steps such as broad outreach, follow-up reminders, and flexible data collection times were taken to ensure equal participation of all the potential respondents. A total of 500 eligible students were approached, of whom 366 participated, yielding a response rate of 73.2%. As this study employed convenience sampling, the findings should be interpreted as representative of the participating students only and not generalized to all medical students. The data collection was performed through a structured self-administered questionnaire in English. The basic demographic portion, including age, gender, medical college, and year of study, was asked in the initial section of questionnaire.

### Assessment tools

To assess the nicotine dependence, the Fagerstrom Test for Nicotine Dependence (FTND), a six item validated scale, was used (Table 1)[17]. The FTND is publicly available and widely used in research without requiring special permission. The FTND provides a continuous score ranging from 0 to 10, based on six items. Three of these items are yes/no questions, scored as 0 (No) and 1 (Yes). The remaining three are multiple-choice questions, scored from 0 to 3. The scores from all six items are summed to calculate the total dependence score. Classification of nicotine dependence: 0–2 (very low), 3–4 (low), 5 (moderate), 6–7 (high), and 8–10 (very high). Reliability analysis showed that the FTND had a Cronbach’s alpha of 0.702, indicating acceptable internal consistency. Nicotine dependence analyses using the Fagerström Test were conducted only among current smokers (*n* = 59), as non-smokers do not engage in nicotine use.

**Table 1:** Fagerström test for nicotine dependence (*n* = 59).

Question	Answer	No.	%
How soon after you wake up do you smoke your first cigarette?	31–60 minutes	9	15.3%
	6–30 minutes	14	23.7%
	After 60 minutes	19	32.3%
	Within 5 minutes	17	28.8%
Do you find it difficult to refrain from smoking in places where it is forbidden (e.g., public transport and hospitals)?	Yes	33	55.9%
	No	26	44.1%
Which cigarette would you hate most to give up?	The first one in the morning	35	59.3%
	All others	24	40.7%
How many cigarettes/day do you smoke?	≤10	32	54.2%
	11–20	13	22.0%
	21–30	5	8.5%
	≥31	9	15.3%
Do you smoke more frequently during the first hours after waking up than during the rest of the day?	Yes	22	37.3%
	No	37	62.7%
Do you smoke if you are so ill that you are in bed most of the day?	Yes	30	50.8%
	No	29	49.2%

The participant of our study were also provided with a subscale, and Family Assessment Device-General Functioning (FAD-GF), with 12 questions to evaluate family functioning (Table 2)[18], was used with explicit written permission obtained from Dr. Gabor I. Keitner, one of the developers of the scale. Each item was provided with a score; greater the score, the worse the family function. Each item was rated on a four-point Likert scale ranging from 1 (strongly agree) to 4 (strongly disagree), with negatively worded items requiring reverse coding to maintain scoring consistency. A cutoff mean score of 2.00 was applied. Scores equal to or exceeding 2.00 are typically indicative of unhealthy or dysfunctional family functioning, while scores below 2.0 reflect relatively healthy family interactions. The FAD-GF scale demonstrated a Cronbach’s alpha of 0.736, also indicating acceptable internal consistency.

**Table 2:** General functioning subscale of family assessment device (*n* = 366).

Question	Answer	No.	%
Planning family activities is difficult because we misunderstand each other.	Strongly agree	38	10.4%
	Agree	131	35.8%
	Disagree	139	38.0%
	Strongly disagree	58	15.8%
In times of crisis, we can turn to each other for support.	Strongly agree	166	45.4%
	Agree	168	45.9%
	Disagree	27	7.4%
	Strongly disagree	5	1.4%
We cannot talk to each other about the sadness we feel.	Strongly agree	49	13.4%
	Agree	153	41.8%
	Disagree	119	32.5%
	Strongly disagree	45	12.3%
Individuals are accepted for what they are.	Strongly agree	55	15.0%
	Agree	193	52.7%
	Disagree	102	15.0%
	Strongly disagree	16	4.4%
We avoid discussing our fears and concerns.	Strongly agree	68	18.6%
	Agree	166	45.4%
	Disagree	105	28.7%
	Strongly disagree	27	7.4%
We can express feelings to each other	Strongly agree	60	16.4%
	Agree	191	52.2%
	Disagree	95	26.0%
	Strongly disagree	20	5.5%
There are lots of bad feelings in the family.	Strongly agree	22	6.0%
	Agree	115	31.4%
	Disagree	172	47.0%
	Strongly disagree	57	15.6%
We feel accepted for what we are.	Strongly agree	54	14.8%
	Agree	215	58.7%
	Disagree	84	23.0%
	Strongly disagree	13	3.6%
Making decisions is a problem for our family.	Strongly agree	28	7.7%
	Agree	112	30.6%
	Disagree	168	45.9%
	Strongly disagree	58	15.8%
We are able to make decisions about how to solve problems.	Strongly agree	64	17.5%
	Agree	232	63.4%
	Disagree	63	17.2%
	Strongly disagree	7	1.9%
We do not get along well together.	Strongly agree	17	4.6%
	Agree	82	22.4%
	Disagree	186	50.8%
	Strongly disagree	81	22.1%
We confide in each other.	Strongly agree	60	16.4%
	Agree	222	60.7%
	Disagree	74	20.2%
	Strongly disagree	10	2.7%

Peer pressure was assessed using the Indicators of Peer Pressure adopted from Subramaniam *et al*[8] (Table 3). Peer pressure was classified as a binary variable based on any affirmative response. We acknowledge that this approach does not capture intensity or frequency and may overestimate prevalence. The questionnaire contains six questions that are divided into direct and indirect peer pressure. The students were asked to answer “yes” or “no” to the questions provided. There were three questions for direct peer pressure and three for indirect peer pressure. Students answering “yes” to any question were considered to have experienced peer pressure and while those who responded “no” to all were seen as free from peer pressure.

**Table 3:** Indicators of peer pressure.

Indicator of peer pressure	Yes		No		
	Frequency	%	Frequency	%	
Peer offer cigarette	104	28.4%	262	71.6%	
Peer ask to smoke with them	92	25.1%	274	74.9%	
Peer force to smoke	43	11.7%	323	88.3%	
Feeling of being avoided by peers if do not smoke	38	10.4%	328	89.6%	
Feel the need to show-off to peer that they smoke	34	9.3%	332	90.7%	
Feel that their peers are “cool” if they smoke	56	15.3%	310	84.7%	

### Statistical analysis

Statistical analysis was carried out using SPSS version 26. Assumptions for normality and homogeneity of variances were assessed prior to conducting parametric tests. Normality was confirmed by visually inspecting bell-shaped histograms and analyzing skewness and kurtosis values, all falling within the acceptable range of ±1.96. Homogeneity of variances was tested using Levene’s test. Descriptive statistics were used to summarize demographic data, smoking status, peer pressure, and family functioning. Two-way ANOVA was conducted to assess the interaction effects of family functioning and peer pressure on nicotine dependence. Due to very small subgroup sizes (e.g., functional families without peer pressure, *n* = 3), the two-way ANOVA results are exploratory and hypothesis-generating rather than definitive. For group comparisons, an independent *t-*test was performed between students with functional and dysfunctional families experiencing peer pressure. A chi-square test was used to test associations between gender, year of study, peer pressure, and smoking status. Assumptions for normality and equal variances were verified before applying parametric tests, and the level of statistical significance was set at *P* < 0.05. This study has been reported in line with the STROCSS criteria[19].

## Results

In this study, 59 (16.1%) of the students were smokers, 153 (41.8%) reported peer pressure, and 293 (80.1%) exhibited dysfunctional family functioning. A significant interaction between family functioning and peer pressure on nicotine dependence was observed [*F*(1, 55) = 4.25, *P* = 0.044]. Males were more likely to smoke and experience peer pressure (*P* < 0.001).

Of the 366 students, 265 (72.4%) were aged 21–24, 89 (24.3%) were 18–20, 7 (1.9%) were under 18, and 5 (1.4%) were 25 or older. There were 175 male students (47.8%) and 191 female students (52.2%). Most of the respondents were from the third year, 154 students (42.1%) in total, followed by 84 students (23.0%) from the fourth year, 57 students (15.6%) from the second year, 36 students (9.8%) from the first year, and 35 students (9.6%) from the final year. Based on the WHO classification, 59 students (16.1%) were current smokers, and 307 (83.9%) were identified as non-smokers. A total of 123 students (33.6%) reported that they had a smoking peer, while 243 (66.4%) reported otherwise (Table 4).

**Table 4:** Demographic characteristics of participants (*n* = 366).

Parameter	Category	No.	%
Age	Under 18	7	1.9%
	18–20	89	24.3%
	21–24	265	72.4%
	25 and above	5	1.4%
Gender	Female	191	52.2%
	Male	175	47.8%
Year of study	First year	36	9.8%
	Second year	57	15.6%
	Third year	154	42.1%
	Fourth year	84	23.0%
	Final year	35	9.6%
Have you smoked cigarettes or any other tobacco products in the past 30 days?	Yes	59	16.1%
	No	307	83.9%
Smoking peer	Yes	123	33.6%
	No	243	66.4%

**Figure 1.**
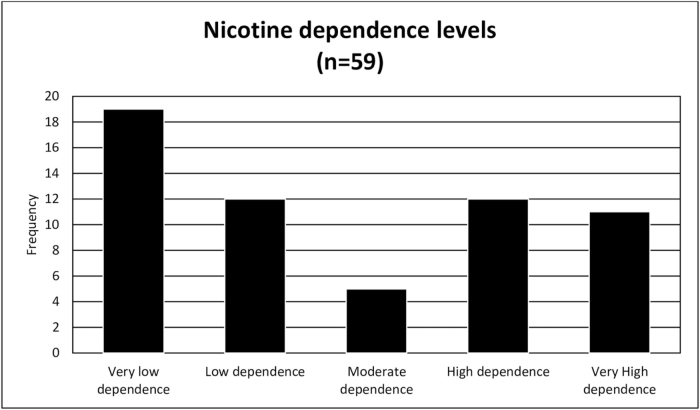
Nicotine dependence levels.

FTND scores indicated that nicotine dependence varied among the 59 smokers from very low to very high, as shown in Figure 1. Although a higher mean FTND score was noted among individuals from functional families without peer pressure, this result was based on a small sample (*n* = 3) and lacked statistical significance. Based on the FAD-GF subscale, 293 students (80.1%) were found to have dysfunctional family functioning, whereas only 73 (19.9%) had functional family functioning. Peer pressure was experienced by 153 students (41.8%), while 213 (58.2%) were unaffected (Table 5).

**Table 5:** Distribution of participants by nicotine dependence level, family functioning (FAD-GF), and peer pressure group.

Variable	Categories	No.	%
Nicotine dependence level (*n* = 59)	Very low dependence	19	32.2%
	Low dependence	12	20.3%
	Moderate dependence	5	8.5%
	High dependence	12	20.3%
	Very high dependence	11	18.6%
FAD-GF category (*n* = 366)	Functional	73	19.9%
	Dysfunctional	293	80.1%
Peer pressure group (*n* = 366)	Absent peer pressure	213	58.2%
	Present peer pressure	153	41.8%

**Figure 2.**
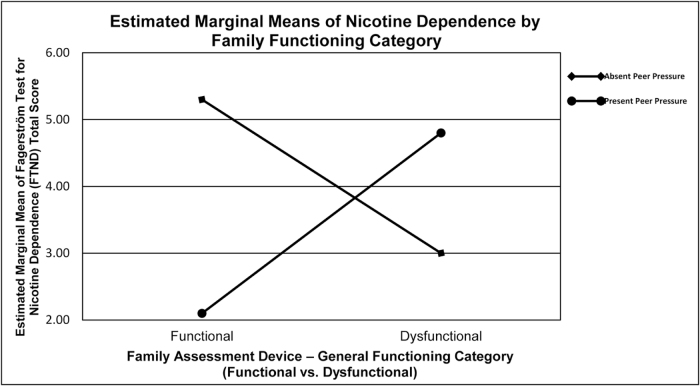
Interaction between family functioning and peer pressure on nicotine dependence (figure descriptive; small subgroup sizes limit inference).

A two-way ANOVA was performed, which showed a significant interaction effect between family functioning and peer pressure on nicotine dependence [*F*(1, 55) = 4.25, *P* = 0.044] as shown in Figure 2. Subsequent analysis revealed no significant difference in nicotine dependence across family functioning groups when peer pressure was absent. However, nicotine dependence was significantly higher in those from dysfunctional families compared to functional families when peer pressure was present (Table 6). This result was further validated by an independent samples *t*-test [*t*(49) = −2.37, *P* = 0.022].

**Table 6:** Mean nicotine dependence scores (FTND) by family functioning category and peer pressure status.

Family functioning	Peer pressure	Mean FTND	No.
Functional	Absent	5 (–)	3
Functional	Present	2 (–)	7
Dysfunctional	Absent	3 (–)	5
Dysfunctional	Present	5 (–)	**44**

A chi-square analysis revealed that males were significantly more likely to smoke [χ^2^(1, *N* = 366) = 25.63, *P* < 0.001) and experience peer pressure [χ^2^(1, *N* = 366) = 48.55, *P* < 0.001). The analysis also showed a significant variation in peer pressure experience by year of study [χ^2^(4, *N* = 366) = 10.90, *P* = 0.028).

## Discussion

Our study investigated the impact of family functioning and peer pressure on nicotine dependence among medical students. The study findings show that 16.1% of the students were smokers, about one-third of them showing a high or very high nicotine dependence. This prevalence is alarming, particularly within a population expected to serve as future healthcare providers and role models for public health. Despite the high prevalence of family dysfunction (80.1%) and peer pressure (41.8%), neither was found to be an independent predictor of nicotine dependence. This study is among the few to highlight a synergistic effect in which peer pressure contributes to nicotine dependence primarily by increasing the vulnerability created by family dysfunction. Specifically, students from dysfunctional families were significantly more prone to nicotine dependence when under peer pressure, a relationship that was not observed in the absence of peer pressure. This suggests that family dysfunction was associated with higher susceptibility to nicotine use in the presence of peer pressure, suggesting a potential interaction between familial and social influences. Additionally, males were significantly more likely to smoke and experience peer pressure, indicating a gender-specific susceptibility.

Previous research has consistently reported a strong and direct relationship between peer pressure and substance use^[^20,21^]^. A study reported that 60% of the students who smoked had been influenced by peer pressure[22], while another study demonstrated that peer pressure in particular had a strong influence on adolescents’ smoking behavior; individuals whose friends smoked were up to six times more likely to smoke[23]. Our study contradicts this pattern among medical students and shows that peer pressure alone was not an independent predictor of nicotine dependence but significantly amplified the effect of family dysfunction through an interaction effect. Higher nicotine dependence was observed among students from dysfunctional families only when peer pressure was present, a relationship that was absent in with put peer influence. A disrupted family environment can lead to individuals with impaired emotional regulation and parental support, increasing their susceptibility to social influences. As a result, their likelihood of engaging in smoking behavior increases with peer pressure[24]. In the context of Pakistani culture, where extended family structures, parental authority, and family reputation play a crucial role, family dysfunction may lead students to depend more on peer groups for emotional and social support, increasing susceptibility to peer influence[25]. In Pakistan’s collectivist cultural setting, coping mechanisms in young adults are molded by high parental expectations, hierarchical parental authority, and social pressures to remain obedient[26]. In situations where families provide limited emotional support, experience ongoing conflict, or have poor communication, students frequently seek acceptance and support from their peer groups[27]. Peer groups in medical colleges often serve as primary social units due to long academic hours, collective stress, and residential life, intensifying peer influence on health behaviors, including smoking[28]. This cultural context helps clarify why the combination of peer pressure and family dysfunction raises students’ susceptibility to developing nicotine dependence in our study. This implies that the link between peer pressure and nicotine dependence is more intricate than earlier studies have indicated, highlighting the combined impact of social and familial factors on smoking behavior in this group.

Family dysfunction and domestic problems showed higher prevalence among the medical students who smoked, but they did not show a marked independent association with the level of nicotine dependence, which, too, contradicts previous literature. For instance, a study shows that a supportive family environment can significantly lower the risk of smoking initiation in adolescents[29], further augmenting our results that reveal that family dysfunction makes young adults more prone to nicotine dependence. Similar to earlier studies, our results suggest that improving the connection of parents with their children is important in preventing smoking habits among young adults[30]. Along with family problems, poor enforcement of age-related restrictions allows adolescents easy access to E-cigarettes; additionally, the lure of flavored options may also encourage the use of tobacco containing products[31].

Our study showed that smoking was more common among male students reflecting the broader gender norms in Pakistan, where smoking among men is normalized, but it is deemed socially inappropriate for women[32]. Although this difference may partly reflect real gender difference in behavior, it is possible that social stigma may have influenced women’s willingness to disclose smoking, resulting in potential underestimation, a possibility that cannot be directly verified in the present study. Although smoking prevalence appeared lower among female students, this may reflect cultural norms and social desirability bias rather than an actual difference. Moreover, they frequently conceal smoking due to social stigma in conservative societies[33]. This potential underestimation aligns with evidence from South Asia indicating women are less likely to disclose tobacco use in household surveys because of cultural expectations and social desirability bias[34]. Future studies using mixed-methods approaches are recommended to better explore unreported smoking behaviors and the sociocultural barriers affecting female smoking disclosure. These gender differences in tobacco use are concerning given its harmful health effects and highlight the need to develop tobacco control policies that address the gender and culture-specific influences on smoking behavior.

To effectively address this matter, institutions must take proactive steps by developing and maintaining student-friendly cessation services that include behavioral counseling, nicotine replacement therapy, and peer support. Tobacco harm education must be integrated into student orientation and the general curriculum, emphasizing long-term health risks and addiction patterns[33]. Successful public policy for student smoking cessation requires multi-layered interventions combining enforcement, education, access to care, and peer engagement[35]. Although family dysfunction was not statistically linked to smoking, engaging families in mental health and wellness programs could still support students’ overall emotional well-being and resilience[36]. Medical institutions have a responsibility to implement educational initiatives focused on smoking cessation, motivational interviewing, and mental health support to prepare clinically capable and peer-supportive professionals.

## Limitations

Some limitations should be acknowledged. Despite involving participants from different medical institutions, the sample might not fully represent all medical students in Pakistan, limiting generalizability of the findings. The limited number of current smokers reduced the statistical power required to detect associations. The reported smoking prevalence might not reflect the actual rate, as smokers frequently comprise a larger portion of those who do not respond[37]. Due to very small subgroup sizes (e.g., functional families without peer pressure, *n* = 3), the two-way ANOVA results are exploratory and hypothesis generating rather than definitive. On the other hand, peer pressure was classified as a binary variable based on any affirmative response. We acknowledge that this approach does not capture intensity or frequency and may overestimate prevalence. Another limitation is that the cross-sectional design of study does not support causal inference, making it difficult to determine the relationship between the measured variables and nicotine dependence. A notable limitation is the inability to exclude potentially influential variables, such as academic stress, mental health, financial status, and access to nicotine, all of which may have shaped nicotine use and dependence. Finally, although we reported nicotine dependence categories, smaller subgroup sizes limited further analysis of family and peer effects.

## Conclusion

This study reveals that peer pressure was associated with higher nicotine dependence among students from dysfunctional families, suggesting a potential interaction effect that requires cautious interpretation. These findings highlight the need for coordinated action at the institutional level to implement smoking cessation programs, counseling services, and risk awareness sessions, with emphasis on gender-specific strategies. If implemented through targeted institutional interventions, these findings could contribute to a meaningful reduction in smoking rates among medical students in Pakistan, potentially decreasing rates by approximately 10%–20% over time. Future research should include additional psychological and environmental contributing factors to deepen the understanding of smoking behaviors among students.

## Data Availability

Data that support the results of this study are available from the corresponding author upon request.
